# Cementless fixation in total knee arthroplasty: current evidence and future perspective

**DOI:** 10.1007/s00402-024-05670-2

**Published:** 2024-12-28

**Authors:** David J. Haslhofer, Nikolaus Kraml, Christian Stadler, Tobias Gotterbarm, Matthias C. Klotz, Antonio Klasan

**Affiliations:** 1 Department for Orthopedics and Traumatology, Kepler University Hospital GmbH, Johannes Kepler University Linz, Krankenhausstrasse 9, 4020 Linz and Altenberger Strasse 69, 4040 Linz, Austria; 2 Marienhospital Soest, Orthopedics and Trauma Surgery, Widumgasse 5, 59494 Soest, Germany; 3 Faculty of Medicine, Johannes Kepler University Linz, Altenberger Strasse 69, 4040 Linz, Austria; 4 Department for Orthopedics and Traumatology, AUVA Graz, Göstinger Straße 24, 8020 Graz, Austria

**Keywords:** Cementless fixation, Total knee arthroplasty, Uncemented fixation, Implant survivorship

## Abstract

**Introduction:**

Cementless fixation plays an increasing role in total knee arthroplasty (TKA). The objective of this review article is to analyze functional outcomes and survivorship of cementless TKA.

**Materials and Methods:**

A comprehensive literature search for studies reviewing the outcome and survivorship of cementless TKA was conducted. This search was based on the PRISMA 2020 guidelines using PubMed, Medline, and Embase. The included studies were screened by two independent observers.

**Results:**

From 2010 to 2022, fifteen studies were included. Eleven studies compared cementless and cemented TKA. Four studies only covered cementless implants. Survivorship and functional outcomes of cementless TKA are at least comparable to those of cemented implants.

**Conclusion:**

With improvement in manufacturing, and surgical tools for more precise delivery, such as robotic assisted TKA and 3D-printed implants, one can expect increase in usage of cementless TKA, due to a more biological fixation, better survivorship, and outcomes.

## Introduction

Through the last years and decades, biomaterials and implants play a bigger and bigger role in medicine. Due to improvements in implant production and safety there are loads of usage possibilities in all kinds of medical fields – orthopedics is one of them [1].

Osteoarthritis, as a chronic musculoskeletal disease, affects approximately 400 million people worldwide and accordingly has a significant socioeconomic impact [2, 3].The treatment of terminal knee osteoarthritis with total knee arthroplasty (TKA) has been the most effective treatment option for more than 40 years [4].It has been shown that the success rate is up to 90% 20 years postoperative [5, 6]. Cemented TKA generate low long-term rates of aseptic loosening, as one of the central complications [7, 8]. Therefore, performing TKA using cemented fixation is reckoned as the reference standard [9].

Recently, cementless fixation has come into increasing focus and leads to higher interest due to a few reasons. TKA is performed increasingly in younger patients, who show a higher activity level and therefore present a higher load on the prosthesis [10–13]. The higher number of young patients include a higher risk of secondary surgery. Cementless fixation in TKA shows a theoretical advantage of a biological fixation, being potentially longer-lasting and initially preserves the native bone stock [14]. Comparatively, cementless fixation in total hip arthroplasty (THA) is the standard of care for acetabular components and for the most part, of femoral components [15]. Theoretical biological advantage of titanium can therefore be utilized to achieve a more biological fixation.

Another advantage of using titanium based implants is its ability for preventing stress shielding [16]. Stress shielding is the result of a mismatch in Young’s modulus of elasticity between implant and bone – a decrease in bone mineral density occurs [17]. Historically, cementless implants showed higher loosening rates, because of its association with stress shielding [18] – with the massive development in implant design and material development, especially the use of uncoated and coated titanium, this seems to be changing [19, 20].

The objective of this review is to analyze mid- and long-term outcomes and survivorship of cementless TKA. It was hypothesized that cementless TKA shows comparable survival rates and functional outcomes as cemented TKA.

## Material and methods

This study was conducted according to the Preferred Reporting Items for Systematic Reviews and Meta-Analyses (PRISMA) 2020 Guidelines [22]. A comprehensive literature search across three electronic databases – MEDLINE, PubMed, and Embase was performed. The following search terms were included: (“total knee arthroplasty” OR “TKA”) AND (“uncemented” OR “cementless”). The capitalized words represent the Boolean operators. Due to continuous improvement of the materials and geometries of the implants, studies prior to 2010 were not considered. Studies published between 01. 01. 2010 and 01. 01. 2022 were included.

**Fig. 1:**
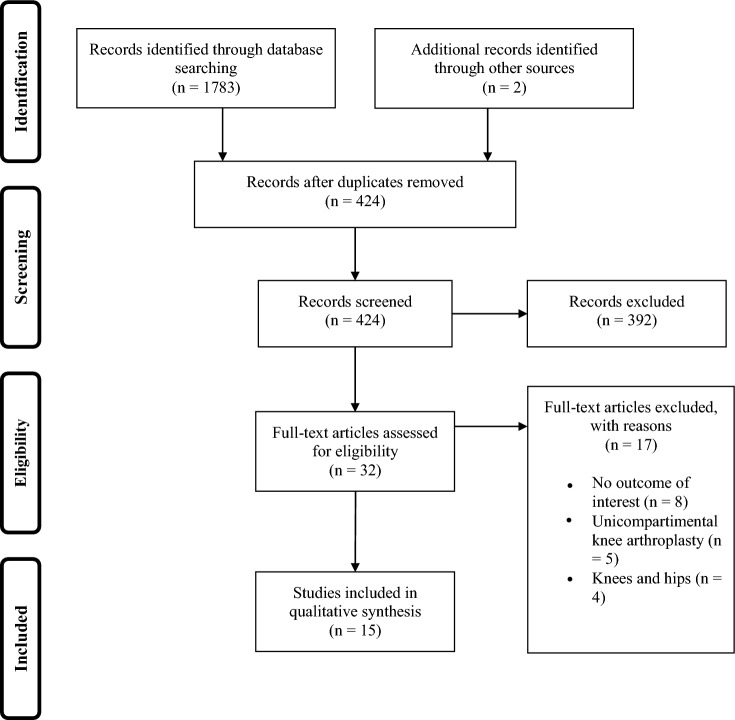
PRISMA Flow Chart

Review inclusion criteria were – 1. primary total knee arthroplasty 2. cementless implants 3. reported implant survivorship, outcomes. For inclusion studies must examine at least 90 patients and have a follow-up of at least 16 months. Studies analyzing cementless unicompartmental knee arthroplasty were not considered. Systematic reviews, conference abstracts, review articles, and expert opinions were not included. Studies without access of full text or studies not in English language were excluded. Additional studies found on this topic have been added manually, Fig. 1.

All studies were imported into Zotero (Fairfax, VI, U.S.) bibliographic software, which was used to remove duplicates. Next, two authors independently reviewed the search results and checked for inclusion. In case of disagreement, a consensus was reached by discussion. In case of discrepancies, a third author was consulted to make the final decision. Afterwards, the studies were searched for the following data: study type, mean age, number of patients (cementless and cemented TKA), follow-up time, outcomes, survivorship, and main findings of the studies. The investigated implants of the included studies are summarized in Table 1.

**Table 1:** Implants

Implants, Company
Triathlon Total Knee System, Stryker Orthopaedics
Vanguard Knee System, Zimmer Biomet
NexGen, Zimmer Biomet
LCS knee arthroplasty, DePuy
TC-Plus Primary; Smith & Nephew Orthopaedics
Multigen, Lima

Furthermore, we accessed the data from the Australian Orthopaedic Association National Joint Replacement Registry, the UK National Joint Registry, and the New Zealand Orthopaedic Association Joint Registry.

## Results

Fifteen studies were included, from the period between 2010 to 2022, Table 2. Seven were prospective cohort studies [23–29] and eight were retrospective cohort studies [19, 30–36]. Comparison between cementless and cemented TKA was performed in 11 of these studies – two of them presented simultaneous treatment in patients [23, 24]. Four studies only covered cementless implants – one of them demonstrated results concerning 3D-printed implants [28].

**Table 2:** Study characteristics

Author	Year of publication	Study type	Implant	Mean age (years)	Number of knees (patients)		Mean follow-up time (months)	Outcomes	Survivorship	Main findings
					Cementless	Cemented				
Park et al. [8]	2011	prospective cohort	simultaneous cementless vs. cemented	58.4	50 (50)	50 (50)	163.2	KSS, WOMAC, ROM, satisfaction score, survival rate	100% both groups femoral components, 100% cemented tibial component, 98% cementless tibial component	No significant differences
Kim et al. [9]	2014	prospective cohort	simultaneous Cementless vs. cemented	54.3	80 (80)	80 (80)	199,2	KSS, ROM, WOMAC, satisfaction score, survival rate	100% both groups femoral components, 100% cemented tibial component, 98.7% cementless tibial component	No significant differences
Fricka et al. [10]	2015	prospective cohort	Cementless vs. cemented	60.2 and 58.6	50 (50)	50 (50)	24	KSS, OKS, pain	97.8% overall survivorship both groups, 97.8% aseptic survivorship cementless, 100% aseptic survivorship cemented	Higher KSS clinical scores for cemented group. Equivalent early follow-up survivorship
Bagsby et al. [15]	2016	retrospective cohort	Cementless vs. cemented	62.7 and 58.8	144 (143)	154 (149)	43.8 and 73.6	KSS, ROM, incidence of revision, aseptic loosening	99.3% cementless PS, 86.5% cemented PS	significantly higher incidence of revision and aseptic loosening in cemented cohort
Behery et al. [16]	2017	retrospective cohort	Cementless vs. cemented tibial fixation	56 and 58	70 (70)	70 (70)	48	complications, morbidity KSS, ROM, SF-12, EQ 5D, UCLA	100% for cemented and 90% for radiographic aseptic tibial loosening	Cementless TKA greater aseptic loosening and revision surgery rate within 5 years follow up. No differences in outcome scores
Bouras et al. [17]	2017	retrospective cohort	Cementless	67.8	136 (113)		158.4	KSCRS	95.7% for aseptic loosening revision at 120 months	high 10- and 15-year component survival rates
Nam et al. [18]	2017	retrospective cohort	Cementless vs. cemented	62.9 and 63.3	66 (66)	62 (62)	16.8	OKS, satisfaction scores	100% overall survivorship both groups, 100% aseptic survivorship both groups	No significant differences
Miller et al. [19]	2018	retrospective cohort	Cementless vs. cemented	64.3 and 64.4	200 (199)	200 (199)	27.6 and 63.4	KSS, ROM, complications	99.5% in cementless and 97.5% in cemented group for aseptic loosening	No significant differences
Choy et al. [11]	2014	prospective cohort	Cementless vs. cemented tibial baseplate	65 and 69	82 (65)	86 (67)	114.9 and 113.8	ROM, HSS, WOMAC, KSS	100% femoral components, 100% both groups tibial components	No significant differences
Stempin et al. [20]	2018	retrospective cohort	Cementless	72.7	106 (99)		66	KSS, WOMAC, aseptic loosening	97.2% overall, 100% for aseptic loosening revision	Good mid-term survival rates
Kamath et al. [12]	2011	prospective cohort	Cementless vs. cemented	N/R	100 (100)	312 (312)	greater 60	KSS	100% cementless group and 99.4% cemented group for aseptic loosening revision	No significant differences
Quispel et al. [21]	2021	retrospective cohort	Cementless vs. cemented	68.4 and 68.5	10,560 (N/R)	190,651 (N/R)	46.2 and 60.4	aseptic loosening, revision surgery	94,5% cemented and 94,2% cementless	Cementless TKA more often revised due to loosening of the components short- and mid-term
Restrepo et al. [13]	2021	prospective cohort	Cementless 3D-printed	63	374 (339)		66	KOOS, VR/SF-12, aseptic loosening, revision surgery	97.06% overall, 98.4% for aseptic loosening	excellent survivorship at mid-term follow-up
Tarazi et al. [22]	2020	retrospective cohort	Cementless	66	228 (228)		minimum 60	ROM, KSS, survivorship	99,5%	excellent clinical outcomes at 5-year minimum follow-up
Lizaur-Utrilla et al. [14]	2014	prospective cohort	Cementless vs. cemented tibial baseplate	51.4 and 52	45 (45)	48 (48)	86.4 and 84	KSS, WOMAC	97.7% cementless group and 91.6% cemented group for aseptic loosening revision	Better clinical outcomes were obtained in the cementless group

*KSS* Knee Society Score, *WOMAC* Western Ontario and McMaster Universities Osteoarthritis Index, *ROM* Range of Motion, *OKS* Oxford Knee Score, *PS *posterior stabilized, *TKA *Total Knee Arthroplasty, *KSCRS* Knee Society Clinical Rating System, *HSS* Hospital for Special Surgery Knee-Rating Scale, *KOOS* Knee Injury and Osteoarthritis Outcome Score, *VR/SF-12* 12-item Veterans RAND/Short Form Health Survey, *EQ-5D-5L *EuroQol-5 Dimension 5-level Instrument, *UCLA* University of California at Los Angeles score

Outcomes were assessed using a variety of scores – Knee Society Score (KSS), Western Ontario and McMaster Universities Osteoarthritis Index (WOMAC), Oxford Knee Score (OKS), Knee Injury and Osteoarthritis Outcome Score (KOOS), University of California at Los Angeles (UCLA) Score, Knee Society Clinical Rating System (KSCRS), Hospital for Special Surgery Knee-Rating Scale (HSS), 12-item Veterans RAND/Short Form Health Survey (VR/SF-12), Range of Motion (ROM), EuroQol-5 Dimension 5-level Instrument (EQ-5D-5L), satisfaction scores, and pain with visual analogue scale (VAS).

Mean follow-up time spanned between 16.8 months in Nam et al. [19] and 199.2 months in Kim et al. [24].

Three studies specifically compared cemented and cementless tibial baseplate with a fixed femoral fixation method [26, 29, 31].

### Survivorship

Survivorship, either overall implant survivorship or data for revision, was presented by all included studies. High mid- to long-term survivorship rates with no statistically significance were presented by most of the studies [19, 23–26, 33]. A few studies could show differences between cementless and cemented TKA.

In a retrospective manner Bagsby et al. [30] compared cementless and cemented TKA in morbidly obese patients (body mass index (BMI) > 40). This study showed a significantly higher incidence of aseptic loosening in the cemented cohort (9 vs. 0 TKA´s, p = 0.005). Overall survivorship reported for cementless posterior stabilized (PS) TKA´s was 99.3% in and 86.5% for cemented PS TKA´s [30]. Two further studies [27, 29] also reported a higher aseptic loosening in the cemented cohort (not significant).

Without presenting detailed implant survivorship rates, Quispel et al. [35] showed a significantly higher revision rate of cementless TKA due to loosening of the tibial (27% vs. 18%; p < 0.001) and the femoral component (7% vs. 5%; p = 0.005) compared to cemented fixation. Overall short- and mid-term revision rates were described similar between cementless and cemented TKA.

Behery et al. [31] also did not present detailed implant survivorship rates but described greater aseptic loosening and revision rates in cementless TKA within 5 years follow-up.

Looking at data of the Australian Orthopaedic Association National Joint Replacement Registry Annual Report 2021 [37] cementless PS TKA´s showed higher revision rates in the short run (< 1.5 years) compared to cemented fixation, and lower revision rates in the long run (> 1.5 years).

The New Zealand Joint Registry 22 Year Report [38] present significantly higher revision rates of uncemented knees than cemented knees. Responsible for these higher rates is the aseptic loosening of the uncemented tibial component [38].

Similar results were revealed in the UK registry data [39]. Primary cementless TKA showed higher revision rates short- and long-term compared to cemented fixation.

### Outcomes

Different outcome scores, especially for functional outcome, were analyzed by 14 of the included studies. Even though comparable outcome data were presented by the majority of the included studies [19, 23, 24, 26, 27, 31], a few differences could be detected.

Higher KSS-clinical scores for the cemented cohort were found by Fricka et al. (92.3 cementless vs. 96.4 cemented; p = 0.03). In this study, KSS functional scores, OKS, ROM, and patient satisfaction showed no significant differences [25].

Bagsby et al. presented out of their patients with BMI > 40 significant improvements in postoperative gained ROM (23.7° cementless vs. 5.7° cemented; p < 0.001), KSS function (26.0 vs. 13.0; p < 0.001), and KSS pain (48.6 vs. 33.3; p < 0.001) in the cementless group [30].

Lizaur-Utrilla et al. [29] reported significantly better postoperative results of ROM (p = 0.042), of KSS score (p = 0.022), and of WOMAC index (p = 0.036) in the cementless group.

Comparing pre- and post-operative KSCRS clinical and functional scores in cementless TKA, Bouras et al. showed a significant improvement in all of these [32]. Stempin et al. [34] reported a significant increase in KSS score and WOMAC index. Also, the study of Tarazi et.al [36] showed a clear increase in the post-operative KSS score.

Better postoperative knee flexion in the cementless cohort (119.4° cementless vs 116.4° cemented; p = 0.003) was found by Miller et al.[33].

The most recent included study, conducted by Restrepo et al., presented significant improvements of KOOS (p < 0.001) and VR/SF-12 (p < 0.001) for their patients treated with a 3D-printed cementless TKA [28].

## Discussion

The most important finding of this systematic review was, that cementless TKA is comparable to cemented TKA in terms of survivorship and functional outcome. Our study reviewed available data on cementless TKA. The average age of a TKA patient is getting lower, the demands are increasing—achieving biological fixation is becoming more and more important.

Although cemented fixation is still the gold standard in TKA, we found that cementless fixation has good performance concerning survivorship rates. Survivorship from 90% up to 100% was detected in cementless TKA. Therefore, cementless data is at least comparable to the cemented fixation data according to the included studies. The Australian Orthopaedic Association National Joint Replacement Registry Annual Report 2021 shows higher survivorship rates for cementless fixation in the long run [37]. However, the New Zealand Joint Registry 22 Year Report and the UK registry data reported higher rates of revision for cementless TKA [38, 39]. One reason for better survivorship of cementless fixation in the studies, compared to the latter register data could be, that in the studies the surgeries were mostly performed by experienced and trained surgeons.

Consideration of bone quality is the most important issue in the decision process of choosing the right fixation – good bone quality is still first requirement in using cementless fixation. In some included studies, patients with inadequate bone stock received cemented fixation instead of a cementless fixation [19, 28, 33]. Intraoperative conversion to cemented fixation is recommend if poor bone quality occurs in patients, whose initial plan of treatment was to get a cementless TKA [40].

Nearly a third of the world´s population is classified overweight [41]. Bagsby et al. [30] compared cementless and cemented TKA in obese patients (BMI > 40). There were significantly more revision and a higher rate of aseptic loosening in the cemented cohort. Sinicrope et al. [42] reported a higher failure rate in the cemented cohort in the same group of patients. Another paper showed similar survivorship between cementless and cemented fixation in obese patients [43]. Due to the large number of overweight patients, larger studies with a longer follow-up are necessary. Perhaps cementless TKA will bring better survivorship in obese patients.

Most of the included studies analyzed the functional outcome using appropriated scores. Data of cementless and cemented TKA are comparable. The majority of the results show no difference between cemented and cementless fixation. Sporadic results were significantly better with the cementless fixation: WOMAC index: Lizauer-Utrilla et al. [29], KSS: Bagsby [30], Miller [33]. However, Fricka et al. [25] reported a significantly higher clinical KSS in the cemented group. All studies could show improvements of all kinds of scores and functions comparing pre- and postoperative. In summary, the same functional outcome can be assumed between cemented and cementless fixation.

In terms of blood loss Parker et al. [23] and Kim et al. [24] presented a significantly higher blood loss using cementless fixation. However, two other studies found no significant difference in blood loss between the two methods [19, 25]. These two papers also reported a significantly less surgical time for cementless TKA. Although the uncemented components are more expensive, the shorter surgery time results in almost the same total costs for both systems [27].

Three studies examined different tibial fixations by either cemented [31] or cementless [26, 29] femoral fixation. All studies analyzed about the same number of patients and had mean follow-up between 48 and 114.9 months. Choy et al. [26] reported for the tibial baseplate no aseptic loosening for both groups and in the study of Lizaur-Utrilla et al. [29] the difference was not statistically significant. However, Behery et al. [31] found a significantly higher incidence of aseptic loosening in the cementless cohort. In addition, more patients in the cementless cohort required a revision surgery compared to the cemented cohort (p = 0.001).

Robotic-assisted arthroplasty as well as 3D-printed implants have the potential to get even better survivorship rates and greater outcomes in the future [44]. Restrepo et al. [28] showed excellent functional outcomes and survivorship of 3D-printed implants. Another study compared functional outcome, complication rates, and revision surgery between cemented and cementless robotic-assisted total knee arthroplasty [45]. Similar outcomes were reported, however the follow-up time was quite short (2 years). These special techniques need to further confirm their improvement status especially in mid- and long-term.

There are some limitations of our study. One of them is that only a comprehensive literature search instead of a systematic review was performed. Another one is that our review does not assess complications in detail. We only focused on survivorship and outcomes. Not covering other cementless knee joint replacements such as unicompartimental implants is also a limitation of our study, but the main focus were TKAs.

Implications: Many studies have already compared cementless with cemented fixation. However, few studies on cementless 3D-printed implants and robotic-assisted TKA are available and no studies, known to us, investigated these two technologies combined. Future research with constantly upcoming new materials, geometries, and technologies will be necessary to get more knowledge about cementless TKA.

## Conclusion

Survivorship of cementless TKA is at least comparable to those of cemented implants. There is also no difference in the functional outcome between cemented and cementless TKA. With improvement in manufacturing, such as additive manufacturing, and surgical tools for more precise delivery, such as robotic assisted TKA and cementless 3D-printed implants, one can expect increase in usage, and potentially, due to a more biological fixation, better survivorship, and outcomes.
